# Translational Potential of MicroRNAs for Preoperative Staging and Prediction of Chemoradiotherapy Response in Rectal Cancer

**DOI:** 10.3390/cancers11101545

**Published:** 2019-10-12

**Authors:** Tana Machackova, Vladimir Prochazka, Zdenek Kala, Ondrej Slaby

**Affiliations:** 1 Department of Molecular Medicine, European Institute of Technology, 625 00 Brno, Czech Republic; tana.machackova@ceitec.muni.cz; 2 Department of Surgery, University Hospital Brno, 625 00 Brno, Czech Republic; Prochazka.Vladimir@fnbrno.cz (V.P.); Kala.Zdenek@fnbrno.cz (Z.K.)

**Keywords:** microRNA, circulating microRNA, biomarker, colorectal cancer, miRNA

## Abstract

Colorectal cancer is the third most common cancer and the second cause of cancer-related deaths. Rectal cancer presents roughly one-third of all colorectal cancer cases and differs from it on both anatomical and molecular levels. While standard treatment of colon cancer patients is radical surgery, rectal cancer is usually treated with pre-operative chemoradiotherapy followed by total mesorectal excision, which requires precise estimation of TNM staging. Unfortunately, stage evaluation is based solely on imaging modalities, and they often do not correlate with postoperative pathological findings. Moreover, approximately half of rectal cancer patients do not respond to such pre-operative therapy, so they are exposed to its toxic effects without any clinical benefit. Thus, biomarkers that could precisely predict pre-operative TNM staging, and especially response to therapy, would significantly advance rectal cancer treatment—but till now, no such biomarker has been identified. In cancer research, microRNAs are emerging biomarkers due to their connection with carcinogenesis and exceptional stability. Circulating miRNAs are promising non-invasive biomarkers that could allow monitoring of a patient throughout the whole therapeutic process. This mini-review aims to summarize the current knowledge on miRNAs and circulating miRNAs involved in the prediction of response to treatment and pre-operative staging in rectal cancer patients.

## 1. Introduction

Colorectal cancer (CRC) accounts for about 10% of all solid tumors and is the third most common cancer worldwide and the second leading cause of cancer-related deaths. Tumors of the rectum—here referred to as “rectal cancer” (RC)—are located up to 15 cm from the anal verge. They comprise approximately 40% of all CRC cases, and approximately half of all RC patients are diagnosed in the stage of locally advanced rectal carcinoma (LARC) [1]. According to the TNM classification, LARCs are tumors in clinical stage II and III (defined as cT3 or cT4) and/or tumours in which the regional lymphatic nodes are affected. LARCs are more likely than colon tumors to relapse locally after surgical treatment, and then metastasize to lungs, liver, and bones [2,3,4]. Since 1980, the incidence of rectal cancer in patients under 40 has quadrupled [5]. Due to high invasiveness and the risk of local recurrence and metastasis, LARC requires different medical management to colon cancer. Usually, LARC treatment is based on neoadjuvant chemoradiotherapy (CRT) with fluoropyrimidines followed by surgical treatment and, eventually, adjuvant chemotherapy. Such an approach aims to improve the local control of the disease and to increase the likelihood of radical surgery with the preservation of the anal sphincter without the need of permanent colostomy. 

Patients with a pathologic complete response (pCR) to therapy have another alternative: They can undergo the “watch and wait” treatment, which implies a non-operative surveillance strategy [6]. In this approach, patients usually undergo CRT, but instead of surgery, the course of their disease is closely monitored. They have to be carefully chosen, however. Patients who might benefit the most from the watch and wait approach include those who are not eligible for surgery, those who wish to avoid abdominoperineal or total mesorectal resection, and those with low stage tumors [7]. 

The watch-and-wait strategy can result in excellent rectal preservation and pelvic tumor control for some of the rectal cancer patients who achieved a pCR [8]. To assess response to therapy, tumor regression grade (TRG) is used to evaluate histologic tumor regression after CRT. Nowadays, several TRG classification systems (e.g., Dworak, Mandard, Ryan, AJCC, Modified Dworak) are used, such diversity additionally complicating precise estimation of tumor regression [9]. Unfortunately, response to treatment varies dramatically between individuals, ranging from pCR to pathologic incomplete response (pIR) to resistance to therapy. Since neoadjuvant CRT is often associated with significant adverse symptoms and high medical costs, these negative effects could be avoided or reduced with the use of biomarkers accurately predicting response to CRT in patients with rectal cancer [10]. 

Experience with other types of cancer suggests that microRNAs (miRNAs) might be such biomarkers. MiRNAs are highly conserved, small, non-coding RNAs, 18–25 nucleotides in length. They act as post-transcriptional regulators of gene expression, by either the post-transcriptional suppression of mRNA translation or induction of mRNA degradation. MiRNA expression is frequently either downregulated or upregulated in tumor tissue versus healthy mucosal tissue, supporting miRNA’s relevance to neoplasia [11]. Another feature that makes miRNAs promising non-invasive biomarkers is their exceptional stability in body fluids [12,13]. 

Given the potential miRNA seem to have to predict CRT response and preoperative staging of rectal cancer patients, this mini-review aims to summarize the current knowledge of miRNAs and circulating miRNAs considered as biomarkers in rectal cancer treatment and discuss their possible use in clinical practice.

## 2. MicroRNAs

The role of miRNAs in cancer still remains unclear. Although little is known about the specific targets and biological functions of miRNA molecules, it is clear that miRNA plays a crucial role in regulating gene expression and controlling diverse cellular and metabolic pathways [14]. MiRNAs are a class of small non-coding RNAs that are 18–25 nucleotides long and function as guide molecules in RNA silencing. They are capable of post-transcriptional regulation of gene expression through the process of RNA interference. At 5’ termini, each miRNA has a sequence that is 2–8 nucleotides long; this sequence is essential for hybridization with their targets. Called the “seed” sequence, it is complementary to the miRNA recognition elements usually embedded within 3’ termini untranslated region of target mRNAs [15]. 

MiRNA genes are localized throughout the whole genome, including intronic, exonic and protein-coding regions. In humans, the majority of miRNAs are encoded within introns of non-coding and coding transcripts. MiRNA genes often occur in clusters and are transcribed as polycistronic transcripts [16]. MiRNA genes usually have their promoters, but if miRNA arises from the protein-coding gene, it can share the promoter of the host gene. According to miRBase assembly version GRCh38, over 2500 mature human miRNAs have been identified so far, each targeting multiple mRNAs and having different effects on different targets [15,17,18]. While the majority of miRNAs are detected in the cellular microenvironment, some miRNAs—so-called circulating miRNAs or extracellular miRNAs—have also been detected in extracellular environments, including many biological fluids (colostrum, breast milk, cerebrospinal fluid, peritoneal fluid, amniotic fluid, synovial fluid, tears, saliva, bronchial lavage, pleural fluid, plasma, serum, follicular fluid, seminal fluid, and urine). Circulating miRNAs occur in the form of complexes with proteins or lipoproteins, which help maintain miRNA stability in the extracellular environments and protect miRNA from degradation by RNases. Circulating miRNAs are present in exosomes, microvesicles, apoptotic bodies, complexes with Argonaut proteins, and complexes with high-density lipoproteins. Transport of circulating miRNAs into extracellular space can be both active and passive [19]. In a recent study, Eslamizadeh et al. observed similar trends in the dysregulation of miRNA in both CRC tissue and CRC plasma, indicating that the expression levels of microRNAs are systematically altered in both of them [20].

## 3. MicroRNAs in Rectal Cancer

Rectal cancer differs from colon cancer at both the anatomical and molecular levels, and several studies have shown that they partially differ in terms of their global miRNA profiles. Tang et al. found that these two types of cancer shared a common global miRNA profile that differed from other gastrointestinal cancers, which share significant similarities in miRNA profiles. This fact is probably caused by midgut/hindgut origins of the colon and the rectum during embryonic development and the reported roles of miRNAs in terminal differentiation [21]. 

Weber et al. compared global miRNA profiles of 15 colon cancer biopsies, 35 rectal cancer biopsies, and control matched adjacent tissues. Using next-generation sequencing (NGS), the authors discovered that two-third of the detected microRNAs had dysregulation specific for either colon or rectal cancer. Only one-third of them had similar expression patterns in both tumor types.

Weber et al. identified miR-133a-3p and miR-375 as miRNAs with the highest potential to distinguish between colon and rectal cancer as well as healthy mucosa. Receiver operative characteristic (ROC) curves of miR-133a-3p showed sensitivity to detect colon cancer with area under curve (AUC) over 0.88 (95% confidence interval [CI] 0.75–1.0). ROC analysis of miR-375 showed sensitivity to detect rectal cancer with AUC of 0.90 (95% CI 0.83–0.97) [22]. According to the literature, the most studied miRNAs with the potential to differentiate between colon and rectal cancers are miR-31 and miR-21. Mir-21 dysregulation was proven to be connected with the pathogenesis of many solid organ tumors, so it is not surprising that this miRNA was found dysregulated in colon cancer and rectal cancer tissues versus healthy colon and rectal tissues [23].

Studies by Wu et al. and Gaedcke et al. showed experimentally that miR-21 expression was significantly higher in rectal tumor tissue than in healthy mucosa. Both studies also evaluated miR-31’s potential as a biomarker in colon and rectal cancers. Its expression was significantly upregulated in RC tissues versus healthy mucosa. Both studies reported that miR-31 was upregulated in rectal cancer versus colon cancer, confirming higher invasiveness and metastatic potential of the former [24,25]. 

Recently, Mu et al. again confirmed significantly higher expression levels of miR-31 in RC tissues than in healthy mucosa. Similarly, RC cell line SW837 showed higher expression of miR-31 than did a human rectal mucosal epithelial cell line. Mir-31 expression was related to distant metastasis, lymph node metastasis, and clinical staging of rectal cancer. Moreover, the authors conducted in vitro functional studies and evaluated miR-31 overexpression and miR-31 inhibition effects on an RC cell line. Cells transfected with miR-31 mimics showed increased invasiveness while cells transfected with miR-31 inhibitor showed decreased invasiveness in comparison to control cells [26]. In the CRC context, miR-31 upregulation was proven to be associated with poor response or resistance of metastatic CRC patients to anti-EGFR (epidermal growth factor receptor) therapy. These observations clearly show that miR-31 plays a significant role in the regulation of RC pathogenesis, through involvement in the regulation of the EGFR pathway, and indicate that carcinomas located in the rectum present malignancy of higher severity than carcinomas located in the colon [27,28,29,30,31]. Yang et al. reported miR-155 to have dysregulated expression in RC and proposed miR-155 as an auxiliary marker for RC tumor staging. ROC curves of miR-155 showed that it can differentiate between N0 and N1-2 stages of RC with AUC of 0.85 (95% CI 0.730–0.980) [32].

## 4. MicroRNAs as Biomarkers of Response to CRT

Administration of neoadjuvant CRT before tumor resection has revolutionized the management of LARC. Nonetheless, many patients are resistant to pre-operative therapy and do not benefit from it. Indeed, response to therapy varies between individuals, ranging from pCR through pathologic incomplete response (pIR) to no pathological response at all. Moreover, pre-operative CRT is often associated with significant adverse symptoms and high medical costs, prompting a need for effective biomarkers of response to CRT. Accurate restaging after neoadjuvant CRT is critical for the optimal planning of surgical treatment. The depth of rectal wall invasion, the presence of nodal metastasis, and the involvement of circumferential resection margin are assessed using pre-operative local staging of rectal cancer, while the presence of metastatic disease is assessed using distant staging. 

Commonly available imaging modalities include transrectal ultrasound (TRUS), computed tomography (CT), magnetic resonance imaging (MRI), and positron emission tomography (PET) [33,34,35]. Pelvic MRI is most widely used for locoregional tumor staging of RC; unfortunately, the reported overall accuracy rates of MRI are about 76% sensitivity and 86% specificity for the assessment of the mesorectal fascia in the irradiated pelvis. Overall, the accuracy of MRI for restaging is generally lower than that for initial staging, mainly owing to the overstaging of nodal disease, failure to differentiate tumoral infiltration or residual tumor from desmoplastic reaction or radiation fibrosis, and the misinterpretation of radiation proctitis as a local invasion [36].

Even though the majority of recent experimental studies on rectal cancer focused on predicting response to CRT, no biomarker have been proposed yet. Hotchi et al. identified miR-233 and miR-142-3p to be differentially expressed between patients classified as responders and non-responders to therapy, based on the histopathological examination of their surgical specimens [37]. Svoboda et al. described different miRNA profiles in responding and non-responding groups of patients. In non-responders, the authors identified three upregulated (miR-215, miR-190b, and miR-92b) and five downregulated (let-7e, miR-196b, miR-450a, miR-450b-5p, and miR-99a) miRNAs [38]. 

Kheirelseid et al. published a study identifying three miRNAs (miR-16, miR-590-5p, and miR-153) with the ability to predict pCR versus pIR to CRT and two miRNAs (miR-519c-3p and miR-561) with the ability to predict good versus poor response to CRT with a median accuracy of 100% [39]. 

Lopes-Ramos et al. reported miR-21 to be differentially expressed between complete and incomplete responders to CRT. Interestingly, miR-21-5p exhibited overexpression in samples of patients with pCR. The considerably low expression levels of miR-21-5p were observed in patients with early local recurrence similarly to patients with pIR [40]. 

In 2016 Caramés et al. published another experimental article investigating miR-31 expression in rectal cancer. Patients who developed pCR had low expression levels while those who had pIR had higher expression levels of miR-31. Additionally, patients with higher miR-31 levels had lower overall survival [41]. Salvi et al. reported dysregulated expression in MIR17HG cluster members. However, only when extreme response classes were compared, miR-19a, miR-19b-1, and miR-92a-1 showed higher levels in TRG0–1 than in TRG4, whereas miR-17, miR-18a, and miR-20a showed lower levels [42]. Millino et al. presented other miRNA profiles corresponding to good or poor response to CRT, with miR-630 being upregulated in non-responder group of patients versus responder group of patients [43]. 

Eriksen et al. showed that the expression of miR-21 above the median expression level was associated with major response to treatment. Patients with miR-125b and miR-145 expression levels below the median had better disease-free survival (DFS) than those with expression levels above the median [44]. D’Angelo et al. identified miR-194 as a potential predictive biomarker of response to CRT [45]. 

Du et al. attempted to determine potential mechanisms of miRNAs and investigated specific miRNA signatures as potential biomarkers for prediction of pCR to CRT in rectal cancer. They detected 36 upregulated (e.g., miR-548c-5p, miR-548d-5p, miR-150-3p, miR-202-3p, and miR-584-5p) and five downregulated (let-7e-5p, miR-1260a, miR-192-3p, miR-26a-5p, miR-30b-5p) miRNAs in the patients with pCR versus pIR group. Additionally, co-regulatory network analysis indicated that miR-548c-5p and miR-548d-5p might function as a complex to co-regulate four genes associated with colorectal neoplasms, namely, IL6ST, CHEK2, MKI67, and MCC [10]. 

Campayo et al. identified miR-21, miR-99b, and miR-375 as CRT response-related miRNAs, all of them having lower expression levels in samples from patients who developed pCR. Two other miRNAs—miR-328 and let-7e—were identified as potential prognostic markers for DFS and OS. ROC curve analysis showed that the combination of miR-21, miR-99b, and miR-375 was the most effective in distinguishing between patients with the maximum response and others [46]. In Luo et al.’s study, miR-519b-3p showed higher expression in patients with pCR than in patients with pIR. The authors also studied in vitro whether this miRNA’s overexpression and inhibition affected therapy response. Using clonogenic assay, they discovered that miR-519b-3p mimics promoted CRC and RC cells sensitivity to chemoradiation treatment while its inhibitors gave the opposite result. Furthermore, after chemoradiation treatment, miR-519b-3p mimics increased cell apoptosis. On the other hand, miR-519b-3p inhibitors decreased cell death [47]. 

Despite so many studies evaluating tissue miRNAs with potential to predict response to CRT, results are highly inconsistent. In fact, not all studies have succeeded to identify miRNAs with such potential, Pettit et al. being one example [48]. The only effect confirmed in several studies is miR-21 upregulation in patients with pCR.

## 5. Circulating MicroRNAs in Rectal Cancer

Circulating miRNAs have been shown outstandingly stable, which makes them very promising non-invasive biomarkers. Liquid biopsy-based biomarkers of response to CRT would enable one to stratify patients before therapy into responders and non-responders. Thus, circulating biomarkers may be a future tool to avoid the exposure to toxic and inefficient treatment for LARC patients with pIR. 

Because of low concentrations in blood plasma and serum, however, circulating miRNA biomarkers are difficult to study. This situation has been changing, thanks to recent advances in biotechnology, in particular improved detection technologies and the widespread availability of kits for the preparation of NGS libraries with RNA low input. The use of NGS increases the chances that new circulating miRNAs will be discovered as potential biomarkers. 

Orosz et al. analyzed the expression of miR-155, miR-21, miR-221, miR-30a, miR-34a, and miR-29a in the blood of patients with colorectal cancer and of control patients, in order to study whether these miRNAs can differentiate between them as well as between colon and rectal cancer. Five out of six of these miRNAs were dysregulated between sera samples from patients with rectal cancer versus those from control patients, two (miR-221 and miR-30a) being upregulated and three (miR-155, miR-34a, and miR-29a) downregulated. Moreover, three miRNAs (miR-21, miR-155, and miR-221) showed different expression levels between colon cancer and rectal cancer [49]. 

Table 1 shows circulating miRNAs that has been shown dysregulated in rectal cancer patients.

## 6. Circulating MicroRNAs and Response to Chemoradiotherapy

D’Angelo et al. identified miR-125b as a potential predictive marker of response to CRT: It showed higher expression levels in both tissue and serum of the non-responder group of patients versus the responder group. This led the authors to hypothesize that miR-125b’s high expression in the tumor tissue influences also its expression in the serum. Furthermore, in the same patients, the Carcinoembryonic Antigen (CEA) serum level before pCRT showed an AUC of 0.5781, too small a value to mark a clear distinction between the two groups [53]. 

Yu et al. found that miR-345 expression correlated with unfavorable response to CRT. MiR-345 expression was significantly downregulated in responders versus non-responders. The authors observed similar results for the expression levels of miR-345 in the tissue of LARC patients. Moreover, they found that lower expression levels of miR-345 positively correlated with local recurrence-free survival. Altogether, these results led the authors to propose miR-345 as a potential individual biomarker of response to CRT in LARC patients [54]. 

Hiyoshi et al. evaluated the potential of 18 circulating miRNAs as predictive biomarkers of CRT response. Only one among them—miR-143—showed significantly higher expression levels in non-responders to CRT versus responders. In addition, the serum miR-143 levels in healthy controls were significantly higher than those in CRT non-responders and responders. No other clinicopathological indicator was associated with a serum miR-143 level [55]. 

Recently, Meltzer et al. reported that poor response to CRT was related to low expression levels of miR-301a-3p and high expression levels of miR-29a-3 [50].

## 7. Circulating MicroRNAs and TNM Staging of Rectal Cancer

Orosz et al., cited above, also evaluated the potential of several miRNAs to distinguish between individual TNM stages. None of them had different expression levels in the sera of TNM I patients and in the control sera. As for the more advanced stages of rectal cancer, the expression levels of miR-155, miR-34a, and miR-29a were downregulated in the sera of TNM II, III, and IV stage patients versus the sera of controls [49]. 

Mjelle et al. provided more complex insight into the global miRNA expression profile in relation to a TNM stage. Using NGS, they identified 26 circulating miRNAs whose expression levels correlated with the TNM stages of the patients. Out them, the authors selected miR-10a-5p, miR-1307-5p, miR-200a-3p, miR-29a-3p, and miR-320d for further qPCR validation. All of them were upregulated in the sera of non-metastatic (TNM I, TNM II, TNM III) versus metastatic patients (TNM IV) [51]. 

Azizian et al. found that the expression levels of circulating miR-18a and miR-20a were connected to the nodal status of patients. In patients with negative postoperative nodal status, the expression levels of miR-18b and miR-20a declined, while in patients with positive postoperative nodal status, the levels practically did not change [52]. 

Meltzer et al. observed elevated expression levels of exosomal miR-141-3p and miR-375 in patients with synchronous liver metastases [55]. In a recent study, Bjørnetrø et al. identified oxygen-sensitive exosomal miRNAs from CRC cell lines and analyzed them in terms of TNM status and treatment outcome. Low expression levels of miR-486-5p and miR-181a-5p were associated with locally advanced disease and nodal metastases while a high expression level of miR-30d-5p was associated with metastatic progression [56]. 

**Figure 1:**
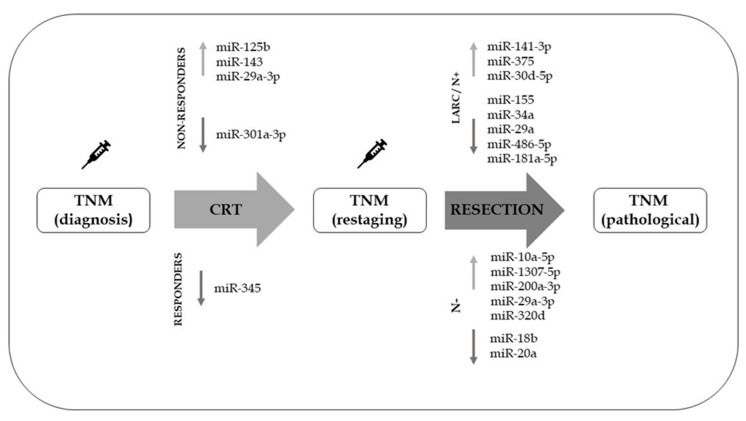
Circulating microRNAs involved in response to chemoradiotherapy or reflecting TNM staging of rectal cancer.

Figure 1 shows miRNAs that have been shown to correlate with TNM staging and response to CRT in rectal cancer.

## 8. Circulating Biomarkers in Rectal Cancer

The invention and development of more sensitive and specific technologies for analyzing circulating biomarkers have advanced research on their use in cancer studies. Circulating biomarkers studied in connection with RC include mainly circulating tumor cells and circulating proteins.

Circulating tumor cells (CTCs) are promising diagnostic biomarkers that allow direct analysis of tumors without invasive intervention. Sun et al. counted CTCs in 115 RC patients and found that patients with TRG 3–4 (Dworak) had fewer post-CRT CTCs than did patients with TRG 0–2 (Dworak). After regrouping the patients according to pCR, the results remained unchanged [57]. 

Flores et al. chose a different approach: in situ hybridization. It enabled them to detect in isolated CTCs two molecules increasing chemotherapy and radiotherapy resistance: thymidylate synthase (TYMS) and excision repair protein RAD23 homolog B (RAD23B). Interestingly, CTCs in all patients with pCR after CRT had undetectable expression of both TYMS and RAD23B [58]. 

Sclafani et al. used digital droplet PCR to detect KRAS (a Kirsten ras oncogene) and BRAF (B-Raf proto-oncogene, serine/threonine kinase) mutations in CTCs. They did not observe any connection between either of the mutations and an RC stage [59]. 

CTCs seem to be promising circulating biomarkers or specimens for further analysis of the response of RC patients to CRT. Research on CTCs will likely give a deeper insight into the mechanisms involved in response to CRT of RC patients, but we should not forget about the one major flaw of CTCs in comparison to miRNA: The isolation of CTCs requires additional processing, which can introduce bias into the analysis. There is no doubt that CTCs cannot compete with miRNAs or other single-molecule biomarkers in terms of stability and simplicity of pre-analytical processing and storing of samples. As is the case with microRNAs, CTCs need to be further studied in larger cohorts of patients since studies performed up to date have shown inconsistent results. 

## 9. Conclusions

MiRNA-based biomarkers have unique advantages: exceptional stability and involvement in the regulation of cancerogenesis. However, to benefit from these advantages, correct pre-analytical processing and storing of samples is critical [60,61]. In CRC clinical practice, the status of KRAS and NRAS (NRAS proto-oncogene, GTPase) mutations serves as a predictive biomarker to anti-EGFR therapy, but is only applicable in the metastatic setting [62]. 

MiR-31 has been widely studied in the context of anti-EFGR therapy response. Its upregulation has been proven to be associated with either poor response or resistance to anti-EGFR therapy in metastatic CRC patients. MiR-31 is thus likely to be implemented as an additional biomarker in CRC, when only its effectiveness is proven in randomized clinical trials. What is more, dysregulation of its expression was reported to correlate with response to CRT. Likewise, its upregulation correlates with poor response to therapy and lower overall survival in RC patients [41]. Nevertheless, miR-31 has low expression in plasma and serum, for which reason it has usually been excluded from validation sets in studies focused on circulating miRNAs in RC. It is for this very reason that miR-31 is unlikely a suitable candidate for a non-invasive biomarker in RC [52]. 

Predictive and prognostic circulating biomarkers in RC make a relatively small field of study, especially against the background of the field of diagnosing CRC. That said, research on this topic is relatively abundant, likely because of the potential such biomarkers show. In the context of RC, naturally, most studies are dedicated to analyzing response to CRT and comparing responders with non-responders. Interestingly, significantly dysregulated miRNAs vary from study to study, including miRNAs that are dysregulated between responders and non-responders. 

Other findings of tissue or circulating miRNAs related to TNM clinical stage or response to CRT have been rather sporadic, and their results require confirmation in studies of various patient cohorts. Two of the reasons behind the inconsistency in the results of different studies may be different definitions of patient groups and different biological specimen used (e.g., formalin fixed paraffin embedded tissue, fresh frozen tissue, tissue in RNA stabilization solution). Different processing and storing of samples can matter, too: These processes affect the overall quality of a sample, thereby affecting the miRNA profile of the sample. The majority of the studies cited above, however, used fresh frozen tissue. 

The most significant differences were observed when comparing IV stage alone with I, II, and III stages. Currently, however, no circulating biomarker can distinguish between early RC stages.

Many studies did not examine global miRNA profiles but instead evaluated miRNAs selected based on literature search, another possible reason behind the inconsistencies of the results. The studies that examined global miRNA profiles de novo used microarray technology. Unfortunately, microarray technology is obsolete and provides only limited information about the comprehensive miRNA profile of a sample. Hybridization microarray technology allows for the analysis of a limited number of miRNAs. Thus, researchers have to choose miRNAs for analysis, a possible source of bias and a possible reason behind unplanned omission of new miRNAs that are related to cancer. 

In contrast, NGS technology has no such limitations and can analyze many miRNAs at the same time. It does not mean it comes without limitations: Data processing, which plays a major role in final results from NGS, is challenging. 

Another problem resulting in the inconsistency in results could be that too few patients were included in the exploratory phases of the studies. Lastly, intratumor heterogeneity probably contributes to the inability to identify tissue microRNAs involved in the pathogenesis of RC and circulating miRNAs; other cell-free biomarkers might have potential to overcome this issue [63]. 

In summary, studies of miRNAs in RC lack proper validation phases and usually only miRNA profiling results are published. Hopefully, the use of large-scale profiling methods, like NGS, and the inclusion of a sufficient number of patients in the discovery and validation phases of biomarker studies will enable the detection of new circulating biomarkers of CRT response and more accurate TNM staging of RC patients. 

## Figures and Tables

**Table 1:** Summary of circulating miRNAs associated with rectal cancer.

miRNA	Change in miRNA Expression/Clinico-Pathological Outcome	Reference
miR-21	upregulated in rectal cancer vs. colon cancer patients	[49]
miR-30a	upregulated in rectal cancer patients vs. healthy controls	[49]
miR-34a miR-29a	downregulated in rectal cancer patients vs. healthy controls	[49]
miR-29a-3p	upregulated in non-responder group of patients vs. responder	[50]
miR-155	downregulated in rectal cancer patients vs. healthy controls, upregulated in rectal cancer vs. colon cancer	[49]
miR-221	upregulated in rectal cancer patients vs. healthy controls, upregulated in rectal cancer vs. colon cancer	[49]
miR-10a-5p miR-1307-5p miR-200a-3p miR-29a-3p miR-320d	upregulated in TNM IV stage patients vs. TNM I, TNM II, TNM II	[51]
miR-18a miR-20b	upregulated in patients with positive nodal status vs. patients with negative nodal status	[52]
miR-125b	upregulated in non-responder group of patients vs. responder	[53]
miR-345	upregulated in non-responder group of patients vs. responder	[54]
miR-143	upregulated in non-responder group of patients vs. responder	[55]
miR-301a-3p	downregulated in non-responder group of patients vs. responder	[50]
miR-141-3p miR-375	upregulated in patients with synchronous liver metastases	[50]
miR-486-5p miR-181a-5p	downregulated in patients with locally advanced disease and node metastases	[56]
miR-30d-5p	upregulated in patients with metastatic progression	[56]
